# The therapeutic attitude in distal radial Salter and Harris type Ⅰ and Ⅱ fractures in children

**Published:** 2010-02-25

**Authors:** G Burnei, S Gavriliu, I Georgescu, C Vlad, I Drăghici, L Hurmuz, D Dan, D Hodorogea

**Affiliations:** ‘M.S. Curie’ Clinical Emergency Hospital for Children, Bucharest Romania

**Keywords:** Salter Harris Ⅰ and Ⅱ fracture, orthopedic reduction, malunion, open reduction

## Abstract

Introduction: Salter Harris Fractures type, especially type Ⅰ and Ⅱ are treated by orthopedic reduction in the emergency room or operating room, under general anesthesia, followed by plaster immobilization. Neglected or incorrectly treated fractures, leading to malunion and radiocarpal subluxations which require surgical procedure.

Purpose: This paper proposes to evaluate the correctly applied orthopedic treatment and the expose of an original surgical technique in case of neglected and incorrectly treated fractures, leading to mal– unions and impediments in the radiocarpal mobility and aesthetics.

Material and Method: we studied a group of 238 children with Salter Harris fractures type Ⅰ and Ⅱ, treated in ‘M.S. Curie’ Emergency Hospital for Children, Bucharest. Out of the studied group, 200 children were treated by orthopedic reduction and immobilization in a plaster device. Malunions present within 38 children due to neglected or mistreated fractures, underwent open reduction with internal osteosynthesis by a technique that avoids violating the growth cartilage. This technique involves making an internal fixation with the radial joint surface in a normal position.

Results: Children receiving proper orthopedic reduction and immobilization in plaster device, 200 patients, were cured after 30–45 days of immobilization, depending on age and joint mobility which were within normal range. The 38 children with malunions underwent surgery to rectify the position of the radial joint surface. Postoperative results were good, proper position of the radiocarpal joint were made during the surgical procedure. The internal fixation is ensured by a transepiphyseal wire and after 30 days of immobilization in a plaster device the patients started the recovery treatment. Radiocarpal joint mobility returned to normal after a variable period of 3 to 6 months, depending on the patient's age.

Conclusions: Salter Harris Ⅰ and Ⅱ fractures are absolute indication for orthopedic treatment, in a matter of emergency, preferably in the operating room under general anesthesia. Verification is necessary between the 7^th^ and 14^th^ day after orthopedic reduction, to avoid malunions. Malunited fractures require surgical intervention after a special technique, avoiding damage to the growth cartilage and radial epiphysis.

## Introduction

The physeal fractures are relatively rare in the case of a newborn child, infant or toddler. The incidence increases between 7 and 15 years of age, when the epiphyseal bone nucleus is developed enough in order to ensure a sufficient bony strength to the epiphysis. The growth cartilage remains between two bony areas with comparatively higher strengths.

**Fig 1:**
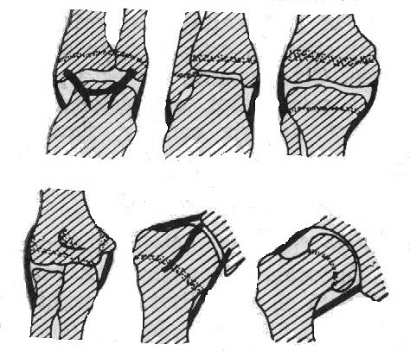
Broca's scheme for the development of physeal fractures.

The force of the trauma applied upon the end of a long bone detaches the epiphysis due to the fact that ligaments resist and actually determine the detachment of the epiphysis. The detachment occurs less often after a direct, epiphyseal injury and more frequently following a combination between traction and torsion exerted indirectly upon the epiphysis.  A long time ago, Broca proved that the detachment occurs in the bone ends, where the capsule and ligaments are joining the epiphysis, as it commonly happens in the lower extremity of the radius, tibia or femur [1] (Fig 1).

This is the place where ligaments cross over the growth plate (i.e. the upper end of the tibia, the hip or the humerus) the growth plate being protected from injuries and fractures which occur only in exceptional cases. The epiphysis is protected at the elbow, while the epitrochlea and epicondyle are frequently detached.

The epiphyseal damage is often accompanied by a small bony fragment of the metaphyseal bone. Sometimes, even a larger part of the bone is detached and, in this case, we call this process fracture–detachment of the physis. These injuries involving the growth plate have been classified by Weber, Poland, Ogden and others, but the most often used classification is the one described by Salter and Harris, based on the altering of the present on the radiological film.

The Salter–Harris classification quantifies the lesion extent of the growth plate, epiphysis and joint. The higher the degree of the lesion, the higher is the chance of angular deformation to develop and the more likely a joint incongruence to appear [2].

**Fig 2:**
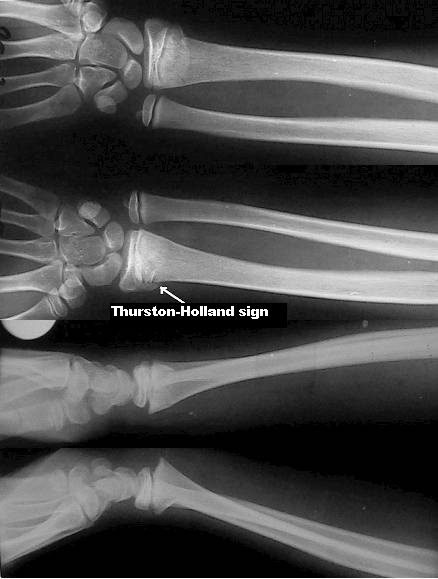
Bilateral radial distal Salter–Harris type Ⅱ fracture (the Thurston–Holland sign)

In type Ⅰ of physeal fracture, the growth plate is detached while the displacement of the fracture's ends may be present or not. In case of type Ⅱ fractures, the detached epiphysis also presents a metaphyseal fragment (the Thurston–Holland sign) (Fig 2) and extremely rare these types of lesions may be followed by physeal arrest [1].

**Fig 3:**
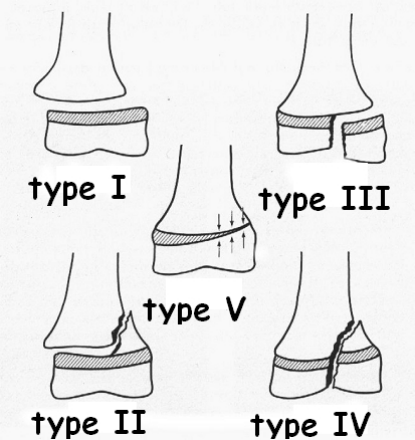
The Salter–Harris classification of physeal fractures

Type Ⅲ consists of the detachment of the growth plate and intraarticular fracture of the epiphysis. The joint is not aligned in case the displacement occurs. Type Ⅳ presents an intraarticular fracture cutting right through the metaphysis, growth plate and epiphysis, the lack of alignment being possible as well. Type Ⅴ can be diagnosed only later and consists of crushing and impaction of the growth plate and a permanent, in this case irreversible damage may occur (Fig 3).

Similarly to other fractures depending on the amount of violence of the trauma, the fragments may remain joined together or may be displaced. Only rarely the epiphysis may undergo a large displacement and the contact with the diaphysis is lost. Usually, there is a certain continuity preserved, but the alignment between the epiphysis and diaphysis may disappear [3]. The periosteal sleeve and joint capsule may resist to the damage impeding the displacement. The lesions involving the growth plate may lead to deficiencies in the further development of the bone and to occurrence of angular deformities.

## Material and methods

This paper presents the results of a series of children with physeal fractures of the forearm bones. The children were either hospitalized or treated in the emergency room. The study period ranges on 10 years, from 1991 to 2000.

The series include 238 children, boys and girls, with ages between 3 and 18 years old (average age 12.5 years old). The sex distribution of the patients showed that the damage was more frequent in boys (30% more for boys than girls).

The forearm physeal fracture diagnosis was established on the clinical and radiological examinations. The anterior–posterior and profile incidence X–ray films included both distal and proximal ends of the forearm. The X–ray films were attentively examined and used to study the location of the post–traumatic lesion.

The patients were subjected to orthopedic reduction in the emergency room or hospitalized and treated by surgical means. Other aspects were also considered in the assessment of the results of the treatment, such as an overall clinical checkup including the esthetical appearance and functionality of the forearm. The results of the treatment were evaluated on X–ray films performed after the treatment was considered finished.

## Results

The sex distribution of the patients in the study group showed the fact that the incidence of the physeal fractures is higher in male compared to female patients. 

The age distribution showed a peak of incidence in the range of 7 to 15 years old. The physeal fractures occur within the cartilage only when the child is very young. The damage takes place in the osteoid area of the plate if the child is a little bit older. In children older than 5 years, the fracture runs through the cancellous part of the metaphysis, right to the border with the diaphyseal–epiphyseal cartilage, and consequently the event is a physeal fracture. A fracture trajectory starts where the detachment stops. The largest majority of our group, 57% of all cases, presented combined lesions of fracture–detachment type, the rest of them (43%) being a pure epiphyseal detachment (Salter–Harris type Ⅰ).

Most often, the location was found in the distal epiphysis of the radius (86%). Other physeal lesions were also met in our group, at all other three epiphyses of the forearm, accompanied by certain types of lesions, as it follows:

- 2 fractures of the radial proximal epiphysis and fracture of the proximal third of the ulna;
- 1 fracture of the radial proximal epiphysis and radio–ulnar distal diastasis;
- 1 ulnar distal epiphysis and fracture of the distal third of the radius;
- 1 double detachment of the proximal epiphyses

**Fig 4:**
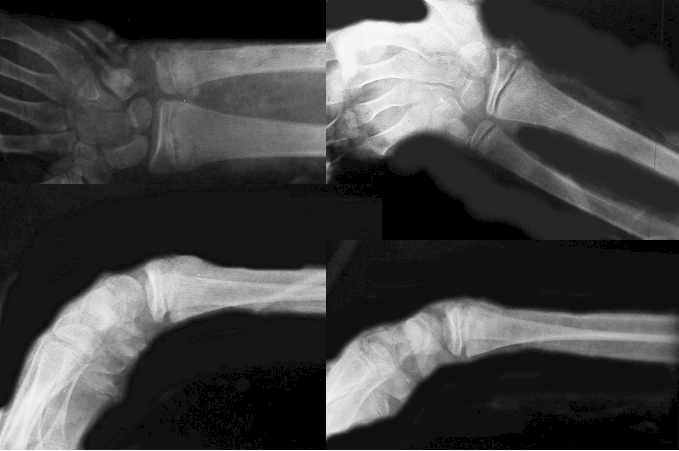
Type Ⅱ Salter–Harris bilateral radial fractures after orthopedic reduction

We performed orthopedic reduction in the emergency room and plaster cast immobilization (Fig 4) in 191 children (80.3%). The rest of 47 children (19.7%) were hospitalized, 38 of them being operated (16%). The other 9 hospitalized were subject to repeated orthopedic reduction under radioscopic control (Table 1).

**Table 1:** The therapeutic attitude relative to the child forearm physeal fractures

Orthopedic reduction in emergency and plaster cast immobilization	191
Hospitalization	Surgical treatment	38
	Orthopedic reduction	9
Total		238

**Fig 5:**
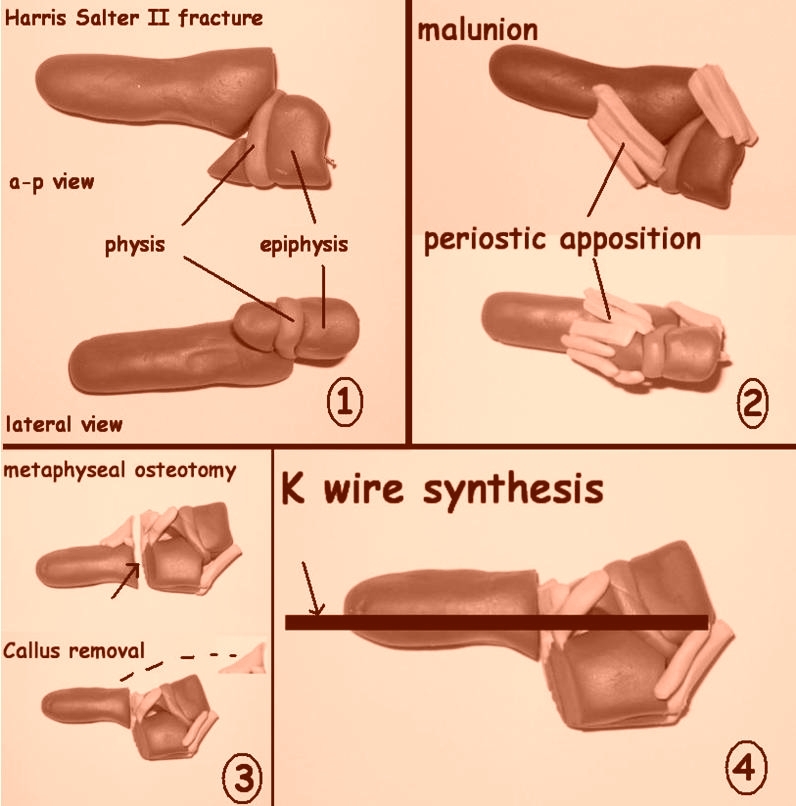
The diagram of the surgical procedure for mal–unions in Salter–Harris type Ⅱ fractures

When surgical approach of malunited fractures is performed, the radio–carpal joint should be checked under fluoroscopic control in order not to damage the physis. Next, a metaphyseal osteotomy is performed and with the aid of a K wire as a guide, the radial joint facet is correctly positioned with lateral and frontal fluoroscopic control so as to avoid joint misalignment inducing late side effects. The defective callus has to be carefully removed starting from the diaphyseal area without periostic apposition, and by no means rasping. The epiphysis has to be fixed manually in the joint by taxis. The osteotomy will be applied to the metaphysis, 0.5 cm below the growth plate. The stability of the radioulnar joint is tested after the proper lining of the joint facet of the radius and epiphyseal fixation with a K wire placed transepiphyseal through the physis into the diaphyseal medullar canal or 2 crossed K wires. The metaphyseal area without bone contact is cut through at the edge of the epiphyseal line and applied to the complementary area to properly direct the joint facet (Fig 5).

The clinical and radiographic examination should be carried out either to receive confirmation or not to neglect the presence of an associated lesion, like the distal radioulnar diastasis, being often met in the distal physeal radial fractures. Its presence leads to pain and restricted prehension power when performing fine movements, as well as for average and high intensity physical activities. If diagnosed early, during the first clinical exam and confirmed by the X–ray films, this lesion allows the performance of a proper orthopedic or surgical treatment.

The plaster cast should be positioned as tight as possible to the radio–carpal joint when orthopedic treatment is applied. The absence of radioulnar diastasis should be confirmed by a supplementary X–ray exam. The plaster cast should be stable and the forearm kept stable after the plaster is removed (using a brassard maintained for an average of three months).

If an operation is needed, the presence of a distal radioulnar diastasis also requires fixation with a percutaneous wire, transversally placed through the radioulnar joint for 3 weeks. The wire will be removed simultaneously with the plaster cast. A persistent diastasis after orthopedic treatment requires pinning of the radioulnar joint in older children and teenagers. Ligamentoplasty is needed in more rebel cases, if prehension abilities are significantly impaired.

We operated 15 children (6.3 %) suffering from distal radial physeal fractures with mal–unions. We performed open reduction and wire synthesis. Two of them needed the cure of diastasis.

The hand mobility was entirely recovered in 13 children, in one case, the result was satisfactory and in one case, the radial deflection of the hand appeared due to a lesion in the growth plate.

A special attention should be paid in case of physeal fractures present earlier than the presence of ossification nuclei of bone. Due to the fact that the ends of epiphyses are inside the joint capsule, the extension force of the brachial triceps exerts directly upon the olecranon, inducing displacements only of variable degrees. We have witnessed this situation in only one case of double disjoining of the proximal epiphyses, in an 8–year–old child. The control proved the detachment of the proximal ulnar epiphysis, too.

The five children with associated lesions and physeal fractures were operated during the first days after trauma. Both lesions were surgically approached with very good results.

The radial deviation of the hand accompanied by the dislocation of the ulnar head, found in 18 children (7.6%),17 of them being operated in other hospitals, were treated with segmental resection of the ulna and osteosynthesis, with the immobilization of the diastasis in 12 children and Darrach approach in 6 children older than 16 years. The results in these cases were good.

## Discussions

The physeal injuries of the forearm are more frequent than in other segments of the limbs. The radial distal epiphysis is more often involved than the ulnar and proximal radial epiphyses.

The further growth evolution may be normal, but deviations are also possible to appear due to asymmetrical lesions of the growth plate. The consequences are linked to the esthetics and function, especially limited hand prehension ability due to restricted or even abolished extension and abduction. These angular deformities occur more frequently in Salter–Harris type Ⅲ, Ⅳ and Ⅴ lesions. 

The majority of type Ⅰ, Ⅱ or Ⅲ Salter–Harris fractures may be treated by orthopedic reduction. It is necessary to use immobilization even if the detachment is not accompanied by displacement, because of the chance of improper growth. The plaster cast should be kept for 15–25 days, according to the child's age and affected bone. The period of immobilization may be shorter compared to other types of fractures because the bone consolidates much faster in these cases.

There is little agreement regarding the acceptable amount of angular deformity present in these types of fractures. Roberts considered that in distal forearm fractures, an up to 35 degrees angular deformities are acceptable [4. Hughston suggested that patients older than 14 years old should be treated like adults, but in children younger than 10 years old a 30 degrees–40 degrees deformity is acceptable [5]. Cooper stated that the acceptable angle is up to 20 degrees [6]. Daruwalla stated that a deformity up to 15 degrees is acceptable and only in children no older than 5 years old [7].

Deformity due to mal–union is the most common complication of distal radial fractures, significant mal–union being sometimes responsible for considerable disability. Up to a 17% rate of mal–union is reported, the rates within non–surgical treatment being higher than those undergoing primary surgery. The results of injury of the distal forearm may lead, to derangement of the distal radioulnar joint and to degenerative changes, besides deformity. This altering may often lead to pain in the wrist and limitation of rotation movement of the forearm with some loss of function [8].

**Fig 6:**
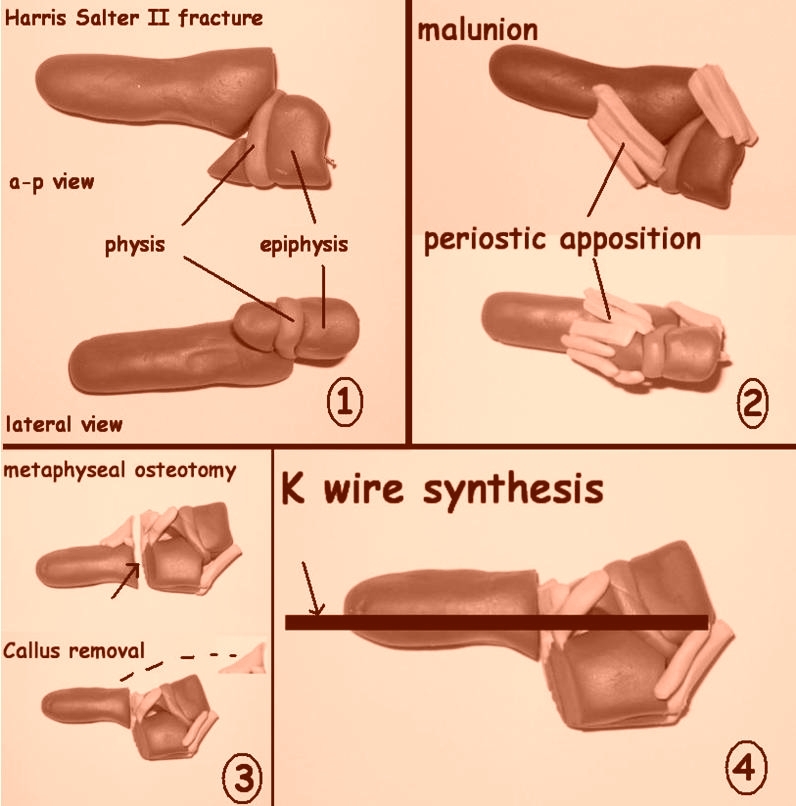
The four radiographic parameters when managing mal–unions of the distal radius.

Bony anatomy is the essential point in the management of mal–unions of the distal radius and it is assessed by four radiographic parameters: radial inclination, radial length, ulnar variance (radioulnar length) and radial tilt (Fig 6).

The radial inclination is represented by the angle between a perpendicular line on the radial axis and a line reflecting the joint surface of the radius. The radial length is represented by the distance between tangential lines to the radial styloid and the ulnar head perpendicular to the axis of the shaft bone. The ulnar variance (radioulnar length) reflects the axial relationship between the ulnar head and the ulnar edge of the lunate fossa of the distal radius. Depending on the length of the ulna, a positive, neutral or negative variance may be described. A more prominent ulna means a more positive value of the variance. The radial tilt (posterior–anterior angularity) is represented by the angle between a line, tangential to the radial joint surface, and a perpendicular one to the shaft axis. The values of these parameters, considered normal, are a radial inclination of 22 degrees (accepted limits ± 15 degrees), a radial length of 11 millimeters (accepted limits ± 4 mm), a neutral ulnar variance (accepted limits ± 15 degrees) and a radial tilt of 11 degrees (accepted limits 15 degrees dorsal and 20 degrees volar). Any changes in these parameters may induce a modified biomechanics of the whole joint system in the proximity of the wrist [8].

Displaced fractures require orthopedic or surgical reduction. The reduction should ensure a parallel alignment of the diaphysis and epiphysis in order to avoid further displacements by an asymmetrical growth. Salter–Harris type Ⅲ and Ⅳ fractures often need surgery and internal fixation in order to put fragments back to the anatomical position. This makes the cartilage grow properly and joint surfaces be congruent. If fractures are not properly treated, fragments will not join, resulting in an angular deformity and a joint misalignment. Type Ⅴ fractures surely present further growth anomalies, regardless of the treatment attitude, because some of the cartilage cells of the growth plate are destroyed.

Not all fractures behave according to this classification, though the Salter–Harris classification is extremely comprehensive. There are cases of Salter–Harris type Ⅰ and Ⅱ fractures that do not evolve positively after the orthopedic reduction and there are cases of types Ⅲ and Ⅳ that do not respond positively to the surgical treatment. It was stated that Salter–Harris types Ⅲ and Ⅳ may undergo orthopedic treatment. Bright remarked that this type of non–displaced fractures may displace during plaster casting. That is why he recommended surgical reduction and internal fixation for all fractures of type Ⅲ and Ⅳ, regardless of the displacement degree [9]. It is advisable to avoid crossing through the growth plate with wires, if possible; wires will cross the epiphysis of the fractured area in types Ⅲ and Ⅳ fractures, while in types Ⅱ and Ⅳ fracture, they will cross the metaphysis and avoid the epiphysis.

Metaphyseal osteotomy is recommended in older children, if a bony bridge and an angular deformity is present. Langenskiold and Bright described a cutting technique of the bony bridge with an interlayer of fatty tissue. Peterson asserts that younger children with an angular deformity under 20 degrees require only the cutting of the bony bridge and corrective osteotomy [9]. Of course, such indications depend on the affected extremity and type of observed deformity. As a rule, a large angular deformity is better tolerated in the thoracic member than in the pelvic member, being more valgus than varus, and flexion than extension.

## Conclusions

The majority of all physeal injuries in the child's forearm may be solved by orthopedic treatment, if diagnosed early and reduced in emergency. Sometimes, a late diagnosis is put in cases with multiple traumas, where abdominal, thoracic and/or cerebral traumatic symptoms are predominant.

The valuation of these fractures should include the distal radioulnar joint as a rule. The presence of the radioulnar diastasis requires an orthopedic or surgical therapeutic attitude.

The surgical treatment of the physeal fractures requires a significant surgical experience to anatomically reduce the fragments and avoid the injury of the growth plate, these issues being very important features for the hereafter function of the child's hand.
